# Dental health of preschool children after two-years of a supervised tooth brushing program in Southern Israel

**DOI:** 10.1186/s13584-021-00479-5

**Published:** 2021-07-22

**Authors:** Lena Natapov, Dan Dekel, Vadim Pikovsky, Shlomo Paul Zusman

**Affiliations:** 1 Dental Health Division, Ministry of Health, Jerusalem, Israel; 2 Ashkelon Regional Health Office, Ministry of Health, Ashkelon, Israel

**Keywords:** Supervised tooth brushing, School dental services, Pre-school dental services, Dmft and its components, Treated fraction of affected teeth, Untreated fraction of affected teeth

## Abstract

**Background:**

Supervised tooth brushing is an important part of leading national oral health improvement programs in different countries.

With the cessation of water fluoridation in 2014, a new program was immediately required to provide community-based caries prevention, especially amongst young children.

The aim of this study was to determine whether a supervised tooth brushing program (STBP) in kindergartens could reduce dental caries amongst preschool children, when compared with children from the same community who did not participate in the program. The study was performed 2 years after the start of the program.

**Methods:**

Two Jewish and two Arab local authorities (one participating and one control) were randomly chosen. In each local authority, 4 kindergartens (children aged 5) were randomly chosen, giving a total of 16 kindergartens. Children in the intervention group brushed once daily at kindergartens, with fluoridated toothpaste, for two school- years. All the children were examined using the WHO Oral Health Survey Methods Ed.4.

**Results:**

Two hundred eighty-three five-year-old children were examined, 157 of them Jewish (86 participants in STBP, 71 non-participants) and 126 Arab (59 vs 67 respectively). Among Jewish children, the fraction of untreated decayed teeth was 61% in the participant group and 65% for non-participants, and amongst the Arab children 69% vs. 90% respectively. The fraction of treated decayed teeth for the participant group was 37% compared to 29% for the non-participants among Jewish children, whilst for the Bedouin group it was 23% vs. 8% respectively.

**Conclusions:**

After 2 years, supervised tooth brushing with fluoride toothpaste shows a favorable effect. This study suggests that dental health of children participating in STBP was better than the control group. Fewer carious teeth and more treated carious lesions were recorded in this group. This program can be applied to low SES communities nationwide.

Guidelines for fluoride concentration in toothpaste for children should be re-considered based on high caries levels.

## Introduction

Until 2014 more than two thirds of the Israeli population was supplied with optimally fluoridated water. Epidemiological surveys carried out in the country have demonstrated the benefits of water fluoridation [1, 2] for dental health and reduction of health inequalities [3]. However, in 2014 fluoridation of water supplies ceased following the new Minister of Health’s decision not to renew the water fluoridation regulations [4]. As a result, a new action plan was immediately needed to continue community-based caries prevention, especially amongst young children.

Dental care for children was included in the National Health Insurance Law in 2010, for children up to age 8. Eligibility age increased gradually to 18 in 2019, providing universal dental care for children.

As a part of dental care reform, community based preventive dental services were extended for preschool and schoolchildren. The School Dental Service (SDS) is described as an effective community-based service for preschool and schoolchildren and includes health education and annual dental screening in kindergartens and schools [5]. The SDS basket of services is wholly state funded. Until 2012, it covered 5-to 14-year-old in compulsory education, in kindergartens, primary and intermediary school settings.

In 2012, free preschool education was extended to include 3–4 year-olds. Following the policy change, health-promoting activities were designed for young children learning at the new settings, emphasizing the importance of healthy nutrition and personal hygiene habits. Dental health promoting activities were included in the daily routine of pre-schools. Daily supervised tooth brushing programs (STBP), are widely recognized as dental caries prevention tool [6–8]. Such a program was started in low SES areas by SDS staff and kindergarten teachers.

SES and lifestyle strongly influence multifactorial diseases such as caries. Southern Israel is generally a low SES area and has Jewish and Bedouin Arabs populations. The Bedouins live in small villages and towns and part of them have a nomadic lifestyle. So, the supervised tooth brushing program was gradually established in preschool settings in low SES areas [9], starting in the Southern Region.

The supervised tooth-brushing program was implemented in 2015–2016 amongst 3–4 year-old children attending 600 nurseries in Israel [10], soon after public water supplies fluoridation was halted [11]. Together with School Dental Service staff, teachers played an important role in program sustainability, enabling and orchestrating activities in classes and supervising children brushing their teeth with fluoride toothpaste [10]. Since 2015, the number of kindergartens participating in the program increased and reached 2200 kindergartens in low socio economic regions nationally in 2019, covering about 15% of preschool settings in the country. The Southern region was the pioneer district, and in 2019, as many as 997 preschool settings belonged to this district.

The objective of the study is to assess dental health indicators such as prevalence proportion of caries, mean dmft and its components as well as treated and untreated fractions of it.

## Materials and methods

### The sample

In 2017, after receiving approval of the Ministry of Health Ethics Committee, a random, cluster, convenient sample was selected in two stages. First, two Jewish and two Arab local authorities (one which participated in the program for 2 years and one control), with similar socio economic characteristics, were randomly chosen for the study. In the second stage, in each authority 4 kindergartens (children aged 5) were randomly chosen, giving a sample of 16 kindergartens out of a total of 320.

### Examination method

The children were examined according to the WHO Oral Health Survey Methods Ed.4. using natural light and a dental mirror. Examination was not performed if a child or his / her parents refused to participate.

One experienced senior researcher working for Ministry of Health performed all the exams.

The data recorded was decayed, missing and filled teeth (dmft). The d component of the index displays the number of teeth affected by caries and untreated. The m refers to teeth missing due to dental caries. The f component refers to teeth affected by decay and restored.

### Statistical analysis

The findings were digitized and analyzed using an SPSSWIN +software. The dmft index was calculated, as well as the prevalence proportion of caries. Unpaired t-test was performed. The fractions of treated (f/dmf) and untreated teeth (d/dmf) out of affected teeth were calculated.

The above mentioned parameters were compared between the two groups of 5 years old preschool children: those who participated in tooth brushing program in the last 2 years, and those who did not by ethnic group.

## Results

Two hundred eighty-three children were examined, 157of them Jewish (86 participants in STBP, 71 non-participants) and 126 Arab (59 vs 67 respectively).

57.3% of the Jews experienced caries vs. 78.6% of the Arab (*p* < 0.0001). The dmft index was 2.3 for the Jewish group and 4.5 for the Arab (*p* < 0.0001), 3.3 for all the children. The d component was 1.4 vs. 3.6 (*p* < 0.0001) respectively as it is detailed in Table 1.

**Table 1:** Dental status by ethnic group

Ethnic Group	N	PP	d	m	f	dmf
**Jews**	157	57.3	1.4	0.1	0.8	2.27
**Arabs**	126	78.6	3.6	0.2	0.71	4.48
Total	283	66.8	2.4	0.2	0.7	3.26

*p* < 0.0001

In Jews the diseased fraction of the dmf (d/dmf) was 70% vs. 84% in Arabs (*p* < 0.001). The treated fraction of the affected teeth (f/dmf) was 29% for the Jews and 13% for the Arab children (*p* < 0.001) as can be seen in Table 2**.**

**Table 2:** Untreated and treated fractions by ethnic group

Ethnic Group	N	d/dmf	Sig. t-Test	f/dmf	Sig. t-Test
**Jews**	90	0.7	*p* < 0.01	0.29	p < 0.01
**Arabs**	99	0.84		0.13	
Total	189	0.78		0.2	

We found differences between the participants and non-participants in both ethnic groups as shown in Tables 3, 5, and 7.

**Table 3:** Dental status by participation

	N	PP	d	m	f	dmft
**participant**	145	62	1.99	0.15	0.86	3.00
**non-participant**	138	72	2.84	0.09	0.60	3.53
		*p* < 0.001	*p* < 0.003	NS	NS	NS

All parameters of dental status were found to be better in the participating students: less prevalence, less decay, less treated disease and lower dmft. The difference in Prevalence Proportion of caries experience between participant and non-participant children was statistically significant (*p* < 0.001). So was the difference in d component as can be seen in Table 3.

The fraction of untreated disease was smaller in the participant children and the fraction of treated disease was higher. These differences were statistically significant as can be seen in Table 4.

**Table 4:** Treated and non-treated fraction by participation

	N	d/dmf	Sig. t-Test	f/dmf	Sig. t-Test
**participant**	90	0.72	*p* < 0.05	0.25	*p* < 0.08
**non-participant**	99	0.82		0.16	
Total	189	0.77		0.2	

Amongst Jewish children, all parameters of dental status were better in the participant than in the non-participant children. 48% of participant Jewish children had caries experience vs 69% of the non-participant, a statistically significant difference, as was the difference in dmft, as it is displayed in Table 5.

**Table 5:** Dental status by participation - Jews

	N	PP	d	m	f	dmft
**participant**	86	48	1.15	0.01	0.71	1.85
**non-participant**	71	69	1.80	0.11	0.85	2.76
**significance**		*p* < 0.007	NS	NS	NS	*p* < 0.007

Fraction of untreated decayed teeth (d/dmf) was 67% in participant group and 72% for non-participants. The fraction of treated decayed teeth (f/dmf) for the participant group was 32% compared to 25% for the non-participants as we can see in Table 6. The situation was found to be better in the participant children, but probably because the lower prevalence, they were not found statistically significant.

**Table 6:** Treated and non-treated fraction by participation – Jews

	N	d/dmf	Sig. t-Test	f/dmf	Sig. t-Test
**participant**	41	0.67	NS	0.32	NS
**non-participant**	49	0.72		0.25	
Total	90	0.70		0.29	

For the Arab group, 83% of the participant Arab children had caries experience vs 75% of the non-participant children. The participant children had significantly higher treatment level (m and f component) as we can see in Table 7.

**Table 7:** Dental status by participation – Arabs

	N	PP	d	m	f	dmft
**participant**	59	83	3.22	0.36	1.07	4.64
**non-participant**	67	75	3.94	0.06	0.34	4.34
**significance**		NS	NS	*p* < 002	*p* < 0.02	NS

The fraction of untreated disease and of treated disease was better in this sector too. d/dmft was 76% vs. 92% while the f/dmf was 31% vs. 19% respectively (*p* < 0.03) as we can see in Table 8.

**Table 8:** Treated and non-treated fraction by participation – Arabs

	N	d/dmf	Sig. t-Test	f/dmf	Sig. t-Test
**participant**	49	0.76	*p* < 0.009	0.31	*p* < 0.03
**non-participant**	50	0.92		0.19	
Total	99	0.84		0.13	

## Discussion

Effectiveness of supervised tooth brushing programs is widely described in the literature: it improves children’s tooth brushing knowledge and behavior and influences parents’ attitudes towards oral health [8, 12, 13]. Regular use of fluoridated toothpaste in tooth brushing programs improves dental health and reduces inequalities and the burden of dental disease [14].

Currently, supervised tooth brushing is an important part of leading national oral health improvement programs in different countries [15, 16]. Numerous studies have shown improvement in dental health of 5-year-olds attending nurseries which implement tooth brushing programs [17, 18]

Yet, “evidence of low certainty” for caries reduction in children was found in a Cochrane library review of community-based oral health interventions (2016). This paper has shown some beneficial effect of supervised tooth brushing with fluoridated toothpaste on dmft and dmfs [19].

In our study, we found a difference in prevalence proportion of caries, in the d and f component as well as in dmft. The differences are statistically significant in PP and d component and treated (f/dmft) and untreated fraction of affected teeth (d/dmft) between children who participated in supervised tooth brushing program in the last 2 years in the kindergartens, comparing to those who did not.

Children from the Arab group, who participated in the supervised tooth brushing program, had less decayed teeth, and much more filled teeth compared to non-participants. These findings show a difference in uptake of dental services within the Arab group, with significantly higher treatment coverage of the tooth brushing group. Even though access to dental treatment has improved dramatically since dental care for children was included into the basket of services of the NHIL, patterns of services uptake are influenced by several factors and differ within the communities. Higher service uptake might have influenced the dmft index in the tooth brushing group, by raised f and m components. However, significantly lower percentage of decayed teeth at age 5 in this group is an important finding, indicating the level of active disease.

Between 2014 and 2016, 600 ppm fluoride toothpaste was used in the kindergarten program. Low fluoride toothpaste (500–900 ppm fluoride) for children aged 2 to 6 years was recommended by national fluoride guidelines established in 2007. These guidelines were issued when about 70% of all water supplies were fluoridated. This policy was implemented in order to prevent dental fluorosis and provide adequate level of prevention from multiple sources of fluoride. Since 2014, when water fluoridation was ceased, much effort has been invested by the professionals, organizations and authorities, to renew fluoridation of water supplies in Israel. The renewal of water fluoridation is highly important because since 2018 most of the drinking water is desalinated water with no microelements in it. In June 2015, the decision to restore fluoridation of drinking water was made by the Ministry of Health, in accordance with the professional opinion of the Ministry’s experts as well as medical associations outside the Ministry. Accordingly, the low fluoride toothpaste policy was left unchanged. However, it has taken longer than expected to restore water fluoridation and the effort is still ongoing, resulting in water supplies in the country lacking adequate fluoride levels for over 5 years.

In 2021 the MoH expert committee advised to update the National Guidelines for Fluoride use in children, according to professional bodies recommendations [20–22] and considering high ECC morbidity. Accordingly, the guidelines were revised and 1000 ppm F toothpastes were recommended for children bellow the age of six. It may be assumed that use of 1000 ppm F Toothpaste in the STB program will endorse the caries reduction effect.

In our study, we found very high caries prevalence among 5-year-olds in both Jewish and Arab Bedouin groups as compared to previous studies conducted in Israel in this age group [4, 5]. Only in one study group (Jewish tooth brushing group), was the caries prevalence less than half (48%). Three other groups of children – Jewish non-participants (PP 69%), Arab non-participants (PP 75%), and Arab participants (PP 83%) - had very high caries prevalence levels.

The anticipated decline in caries levels due to the free of charge preventive dental treatment in basket of dental services of NHIL has not yet occurred. The tooth brushing group of children had better dental health indicators and lower untreated disease, as mentioned above.

### Limitations of the study

dmft index itself is not a sensitive enough index in order to reflect the complexity of oral health status nor severity changes in it.

## Conclusion

Dental caries was highly prevalent among the five-year-old Israeli children examined in this survey and the disease levels remained high compared to previous epidemiological studies for this age group, probably due to the cessation of water fluoridation. Untreated dental disease levels were lower in tooth brushing groups, both for Jewish and Arab children. After 2 years, the STBP shows a favorable effect. Dental health of children participating in the STBP was better. Fewer carious teeth and more treated carious lesions were recorded in this group.

### Policy implications

The high caries level found in children in our study and the favorable effect of STB have policy implications:

- Considering high disease level among children, the national caries prevention policy must relate to various fluoride sources and promote their synergistic effect. Thus, community water fluoridation renewal is an important issue too for policy-makers to address, together with other oral health promotion interventions countrywide.
- Guidelines for fluoride concentration in toothpaste for children were re-considered based on high caries levels.
- Targeted programs, such as supervised tooth brushing and fluoride varnish programs, should be extended nationwide.

## Data Availability

The datasets analyzed during the current study are available from the corresponding author on reasonable request.
